# Toward transparent intelligence: Explainable stacked ensembles learning for LiDAR point cloud segmentation

**DOI:** 10.1371/journal.pone.0345125

**Published:** 2026-05-20

**Authors:** Afridi Rahman Bondhon, Emon Kumar Dey

**Affiliations:** Institute of Information Technology, University of Dhaka, Dhaka, Bangladesh; National Changhua University of Education, TAIWAN

## Abstract

Segmentation of LiDAR point cloud data has various applications, ranging from urban planning to environmental monitoring. Although machine learning approaches have achieved impressive segmentation performance, their black-box nature often limits their interpretability. While stacked ensemble learning improves segmentation accuracy by combining multiple classifiers, it further increases model complexity and obscures decision transparency. To address this gap, this study proposes an explainable stacked ensemble framework for LiDAR point cloud segmentation that integrates multiple base learners with Logistic Regression as a meta-model and incorporates model-agnostic Explainable Artificial Intelligence (XAI) techniques. Experimental results on two benchmark datasets demonstrate segmentation accuracies of 91.13% and 95.71%. The study also identifies the most effective base models within the ensemble to facilitate optimal model selection. Furthermore, XAI-driven feature analysis enables effective feature reduction, achieving a minimum 7% reduction in training time while maintaining consistent accuracy. In addition, variants of the SHAP algorithm are employed to investigate the relevance of features and the impact of neighborhood selection strategies on the segmentation performance of each base model. The experimental results demonstrate that the proposed approach achieves competitive segmentation performance while improving interpretability and computational efficiency.

## 1 Introduction

Point Cloud is a collection of three-dimensional (3D) data points where each point is defined by the X, Y, and Z Cartesian coordinates. Additional attributes, such as RGB color information and surface normals, can often be observed in the point cloud data. Aerial laser scanning (ALS) and terrestrial laser scanning (TLS) are the most common approaches to acquiring highly precise point cloud data based on the Light Detection and Ranging (LiDAR) technology, where the Cartesian coordinates represent the latitude, longitude, and elevation, respectively. A wide range of sites, including urban environments, natural landscapes, agricultural lands, mining areas, disaster-affected zones, and cultural heritage sites, can be captured using ALS and TLS LiDAR point cloud technology.

The direct segmentation of individual point cloud data is a key area of research in photogrammetry, computer vision, and remote sensing. It is the process of identifying or classifying each point based on some local and global features of the input point cloud data. Geometric rule-based, machine learning-based, and deep learning-based segmentation approaches exist in the literature [1].

The machine learning-based methods extract geometric features manually for each point, then use these features to train and test a supervised classifier, such as Random Forest, Support Vector Machine, K-Nearest Neighbors, and XGBoost [2]. Features are generally extracted automatically from the hidden layers of neural networks in deep learning-based methods. Along with the automatically extracted features, geometric features have also been used in many deep learning-based approaches [3,4].

Traditional machine learning-based models require a higher number of suitable geometric features for an enhanced segmentation performance. However, simply adding more features does not always guarantee better performance; rather, it may raise the risk of the overfitting problem [5]. To address these issues, different minimal and robust subsets of feature selection approaches exist in the literature aiming to improve the accuracy and computation efficiency [4]. Moreover, machine learning-based models often operate as black boxes because they cannot explain precisely for which geometric features the models are performing well, also the class-based impact of the selected features cannot be interpreted by the existing machine learning-based point cloud data segmentation models.

To address these critical issues in LiDAR point cloud data segmentation, Explainable Artificial Intelligence (XAI) techniques have recently been introduced to provide more interpretable and trustworthy machine learning models [4]. By examining different attributes of the selected features, such as the biasness, validation of decisions, and counterfactual explanations, XAI algorithms can provide important insights into the internal working procedure of different machine learning segmentation models [6]. These algorithms can tell specifically which features are significant for which class in the final segmentation results, also how and why specific features in 3D data perform accordingly. Thus, the models become more transparent and achieve more trust. This understanding is essential for maintaining accuracy and ensuring the reliability of any automated systems or applications [4].

Based on the strong explanation of the different XAI algorithms, proper features for segmenting LiDAR point cloud data can be selected for a specific machine learning algorithm; however, the selected models and methods often suffer from weak noise resistance of features, handling with outliers, and local optima [7]. To solve these problems, multiple machine learning models are combined to improve the performance of overall segmentation, which is comparatively a new strategy in LiDAR point cloud data processing and known as ensemble learning [7]. It applies stacking to the outputs of multiple models and feeds them into a meta-model.

Although ensemble learning can significantly improve the segmentation accuracy of LiDAR point cloud data by combining multiple models, the outcome is influenced by several base models used and a meta-model. Thus, the interpretability of an ensembled model becomes challenging due to the complexity of model interactions. Standard XAI tools like SHAP or LIME can struggle to explain such architectures, as it requires explaining the behavior of a model of models.

Despite recent progress in LiDAR point cloud segmentation, most existing approaches provide limited interpretability, particularly in explaining class-wise feature contributions and the interactions within ensemble models. While XAI methods have been applied to individual classifiers, their extension to stacked ensemble architectures remains largely unexplored, leaving a critical gap in interpretable and minority-aware LiDAR segmentation.

This study aims to enhance the segmentation performance of LiDAR point cloud data by employing a stacked ensemble learning approach, where multiple base machine learning models are combined. Further, finding the feature importance of each base model within the stacked architecture and then the feature importance of the meta-model using the model-agnostic XAI methods tailored for ensembles. In addition, the contribution of each base model to the architecture is quantified, highlighting its relative importance in the overall decision-making process.

The specific contributions of this work are as follows:

- This study employs a stacked ensemble learning approach for LiDAR point cloud segmentation, combining multiple base machine learning models with Logistic Regression as the meta-model to enhance overall segmentation accuracy and demonstrating the power of combining multiple machine learning models.
- This work utilizes model-agnostic XAI algorithms to evaluate feature importance within the stacked ensemble architecture. By carefully analyzing the contribution of each base model and its selected features, the study offers deeper insights into the model’s decision-making process, improving the interpretability and trustworthiness of the model.
- In addition to feature importance analysis, the study identifies the most effective base model within the stacked ensemble, allowing for a more nuanced understanding of model behavior. This contribution aids in selecting the optimal model configuration for LiDAR point cloud data segmentation.
- Using the variants of the SHAP algorithm, this study investigates their performance in selecting appropriate geometric features for the individual base model. It also highlights the importance of adjusting the neighborhood selection for individual points to improve model efficiency.

The rest of the paper is organised as follows. Section 2 reviews the status of the current research on LiDAR point cloud data segmentation along with the application of XAI algorithms. The proposed methodology of this research is presented and discussed in Section 3. Section 4 represents the experimental results and discussion, and finally, Section 5 concludes this work.

## 2 Literature review

LiDAR point cloud segmentation is the partitioning of the 3D input data by assigning a calculated class label to each point. It facilitates the identification and separation of individual objects within the scanned environment.

Neighborhood selection is a fundamental step in some segmentation methods. Different methods regarding neighborhood selection has been introduced. Elong et al. [8] integrated curvature into the neighborhood search by classifying points based on curvature. They applied the k-nearest neighbor approach to recover neighborhoods in sparse areas and used a multiscale spherical neighborhood technique to extract features from more regular points. Cao et al. [9] introduced a local density-based method for neighborhood recovery. Skrodzki and Zimmermann [10] incorporated the angle between normal vectors of points into the neighborhood selection, assigning varying weights to each point to increase the reliability of the neighborhood. Günen [11] proposed a neighborhood recovery method based on omnivariance, which showed improved classification performance across different datasets. The neighboring points are used to compute features, which are subsequently input into segmentation algorithms to partition the data.

Among the existing approaches of segmentation, the rule-based approach considers a set of “if-then” rules derived from the training data. Martínez et al. [12] used a rule-based segmentation to categorize buildings, vegetation, and ground from LiDAR point cloud data based on a region-growing technique. Awrangjeb et al. [13] used this approach to extract building roof planes. Point-based classification, another form of rule-based segmentation, was utilized by Yastikli et al. [14] to distinguish between classes. However, the deterministic nature of rule-based approaches restricts their ability to model intricate associations between different classes.

Supervised machine learning-based approaches demonstrate robust segmentation performance in aerial LiDAR point-cloud data. By analyzing the computationally derived geometric or spectral feature sets, machine learning algorithms effectively capture hierarchical spatial relationships, facilitating precise semantic segmentation of urban or natural landscapes. Günen [11] used 21 different geometric features for scattered point cloud data segmentation. Li et al. [15] calculated features from a voxel and color fusion was included for better accuracy. Atik et al. [16] tested 8 different machine learning models for multi-class segmentation on 3 different datasets. Chakraborty et al. [1] used several geometric features considering the variation of point cloud density over the input point cloud data. Most of the authors in the literature used mainly Logistic Regression, Linear Discriminant Analysis, K-Nearest Neighbors, Decision Tree Classifier, Naïve Bayes, Multilayer Perceptron, Adaboost, Random Forest, and Support Vector Machines to analyze the results and their corresponding impact [2,17].

Deep learning has emerged as a prominent methodology for point cloud segmentation, leveraging hierarchical neural networks to autonomously extract intricate features and patterns from raw 3D data [18]. The multi-layered architectures of deep learning enable the automatic learning of discriminative representations, eliminating the need for manual feature engineering and human interaction while achieving state-of-the-art performance in various segmentation tasks on complex and large data [19]. Some of the literature used direct raw LiDAR point cloud data for segmentation. PointNet is one of the first approaches in this case, which was introduced by Qi et al. [20]. Later, PointNet++ [21], PointCNN [22], RSNet [23], SO-Net [24], and more used similar direct segmentation approaches using deep learning. Irregular structure of point cloud restricts the relation between points and hinders the deep learning networks to learn correctly [25]. To address the challenges, volumetric deep learning methods were introduced, where point clouds are transformed into a regular volumetric occupancy grid, also known as a voxel. This allows for the effective training of deep learning models on structured data. Some of the popular volumetric methods are VoxNet [26], SEGCloud [27], PointGrid [28]. Deep learning methods automatically extract features compared to machine learning-based segmentation. However, it requires extensive data and computational resources, including long training times, and poses challenges in parameter tuning and optimization [7].

Different multi-view-based deep learning methods are introduced where the 3D data was transformed into 2D data to train the models. Notable examples include multi-view convolutional neural network (MVCNN) [29], SnapNet [30], and SnapNet-R [31]. Hu et al. [32] proposed RandLA-Net, a lightweight neural architecture, for large-scale point cloud processing that can process approximately 1 million points in one pass. Atik and Duran [33] included geometric features and spectral features as input to RandLA-Net to conduct a study measuring the impact of features. Kurdi et al. [34] developed a deep neural network-based model and used 11 features for the segmentation task. Ozturk et al. [35] proposed a fusion of 2D image and 3D LiDAR data for DL-based classification to improve accuracy.

Even though deep learning approaches stand out with outstanding results, processing of large point cloud data with deep learning requires powerful hardware specifications. Moreover, many machine learning and deep learning models operate as a black-box to some extent, and their decision-making processes, particularly in feature importance, are not transparent. This lack of interpretability has led to the adoption of explainable artificial intelligence (XAI) techniques, which enhance transparency and human understanding of the segmentation of aerial LiDAR point cloud data. LIME (Local Interpretable Model-agnostic Explanations), proposed by Ribeiro et al. [36], is one of the first model-agnostic techniques that offers an explanation for classifier predictions in an interpretable and trustworthy manner. The inability of LIME to effectively handle irregular and unordered 3D data has led to the exploration of alternative post-hoc explainability techniques. Among them, SHAP (SHapley Additive exPlanations), introduced by Lundberg and Lee [37], has gained prominence due to its solid theoretical foundation and reliable feature attribution. Linear SHAP, Kernel SHAP, and Deep SHAP are some variants of SHAP. Linear SHAP can interpret linear models efficiently by computing Shapley values analytically. Kernel SHAP is computationally expensive and uses a weighted linear regression technique to calculate the Shapley values. Deep SHAP can calculate Shapley values for deep learning models by combining a backpropagation-based method, DeepLIFT, and the Shapley equation [38]. It is faster than Kernel SHAP for deep learning networks. Ekanayake et al. [39] developed Tree SHAP, which is another variant of SHAP that can explain the feature importance in the tree-based models, such as decision trees, random forests, XGBoost, and LightGBM.

Despite the fact that XAI has recently been used in different segmentation models, these studies have predominantly focused on object classifications. To explain the features using XAI techniques in machine learning-based photogrammetric aerial LiDAR point cloud data segmentation, there is only one paper in the literature, proposed by [4]. However, there are several critical gaps that remain unaddressed in the explainability of different features. They relied exclusively on TreeSHAP, neglecting other advanced and variant XAI techniques, such as Kernel SHAP and Gradient SHAP. They only considered tree-based classifiers. Moreover, they considered a fixed radius neighborhood selection technique, which works well when the input LiDAR point cloud data is uniformly distributed. Thus, they are unable to acknowledge how explanations may degrade under the extracted features in various-density aerial point cloud data. The next section proposes a methodology considering the mentioned research gap in the literature.

Wu et al. [7] in their study discussed different approaches of point cloud segmentation and compared their respective advantages and limitations. They proposed an ensemble model to segment the LiDAR point cloud data by integrating multiple machine learning models and proved that an ensemble model can improve the segmentation accuracy by leveraging the strengths of different base models. However, the explainability of ensemble machine learning models remains an unexplored area in the context of LiDAR point cloud segmentation, warranting further investigation.

## 3 Materials and methodology

**Fig 1:**
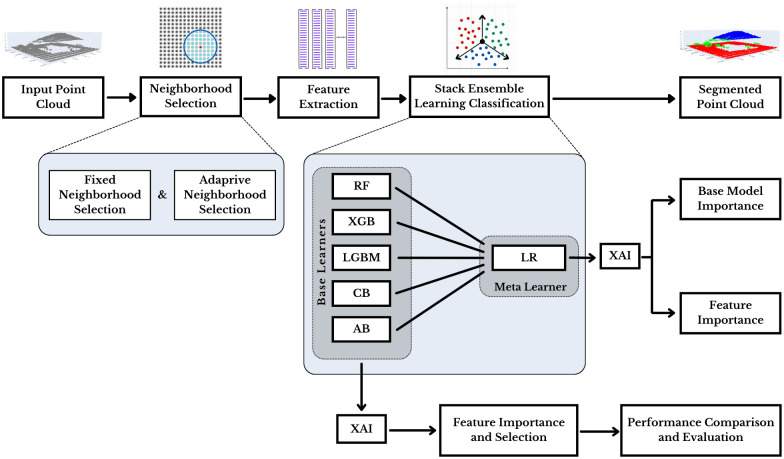
Overview of the proposed methodology.

The foundational methodology of this study is presented in Fig 1. The neighborhood selection process of individual points in the input LiDAR point cloud serves as a basis for feature extraction. The stacked ensemble learning algorithm then leverages base models and learn accurately from these features to segment individual point clouds.

In this architecture, initially, the base models learn from the training data and generate predictions in the form of labels, confidence scores, and posterior probabilities. These outputs are then passed to the meta-model, which uses them to refine the final predictions, capitalizing on the strengths of the base models and mitigating their individual weaknesses. This process enhances the model’s overall efficiency and robustness.

Explainable AI (XAI) methods are employed in two distinct ways within the architecture: First, XAI algorithms are applied to the ensemble’s meta-model to analyze the importance of the base learners’ contribution for this architecture. This helps identify which base models contribute most to the final decision for each class. Second, the input features are mapped to the meta-model’s XAI to find the feature importance. This demonstrates how each feature contributes to the decision-making process.

For further explanations, the XAI algorithm is individually implemented on the classifiers used as the base learner. Feature importance is identified, and the performances of different selected features based on their importance are demonstrated using different evaluation metrics.

The following subsections describe the materials and methodology accordingly.

### 3.1 Dataset

**Fig 2:**
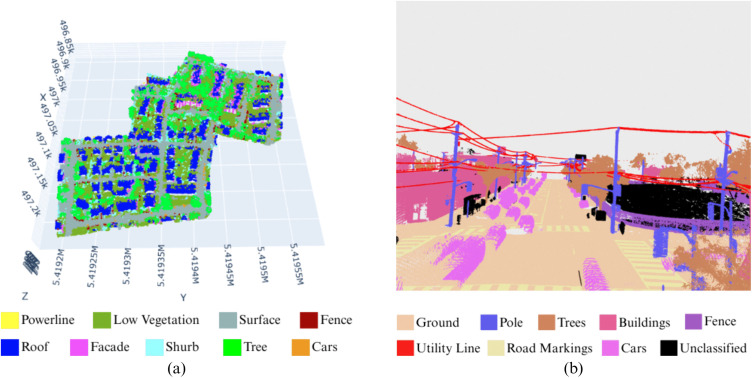
The dataset used in this study. **(a)** Vaihingen Dataset **(b)** Toronto3D Dataset.

This study utilizes both low-density aerial and high-density mobile LiDAR point cloud datasets. The ISPRS Vaihingen laser scanning 3D dataset consists of multiple object classes [40], and the Toronto3D dataset comprises mobile LiDAR point clouds captured along urban road corridors in Toronto, Canada [41]. Both datasets are publicly available and widely adopted in the remote sensing community for evaluating 3D point cloud analysis algorithms. The Fig 2 visualizes the (a) Vaihingen dataset and the (b) Toronto3D dataset.

The ISPRS Vaihingen datasets used an aerial Leica ALS50 scanner from an altitude of 500m at an angle of 45° to collect the data. The data is stored in a ply file format consisting of nine different classes: powerline, low vegetation, surface, car, fence, roof, facade, shrub, and tree. It contains three separate sites with a total of 411,722 points in the two test sites and 753,876 points in one training site. The point density of the dataset is not evenly distributed, ranging from 4 to 8 points/meter^2^ [42].

The Teledyne Optech Maverick scanner was used to capture the Toronto3D dataset. The Toronto dataset contains four different ply files denoted as L001, L002, L003, and L004. They altogether contain a total of approximately 78.3 million points with a point density of approximately 1000 points/meter^2^. It also contains color information for each point. The dataset contains a total of nine classes, including ground, road markings, trees, buildings, powerlines, electrical poles, cars, fences, and unclassified points.

### 3.2 Neighborhood selection

Neighborhood selection is the process of identifying and selecting a subset of points that are considered neighbors of a given point of interest when calculating local geometric features. Selecting appropriate neighbors is important as it determines how efficiently geometric properties are computed. Effective feature calculation enables higher accuracy and robustness of a system [43]. So, neighborhood selection is a fundamental step in point cloud segmentation tasks. There are different neighborhood selection methods for point cloud processing. In this study, the explainability of different features is demonstrated for both fixed radius-based and adaptive neighborhood-based features.

#### 3.2.1 Fixed radius neighborhood selection.

The neighboring points are considered based on a fixed radius ε from a given center of attention point [44]. Let’s take a given point *P*_*i*_, and the given radius is ε. A point *P*_*j*_ will be the neighbor of *P*_*i*_ if and only if the Euclidean distance ‖d‖ between these points is less than or equal to ε.


$$\begin{array}{c} {𝒩}_{\varepsilon }(P_{i})=\{P_{j}\in P\;|\;\|d\|\leq \varepsilon \} \end{array}$$ (1)

Here *P* is the set of all points in the point cloud and ${𝒩}_{\varepsilon }(P_{i})$ denotes the neighboring points of point *P*_*i*_. The larger the radius we take, the more points will be considered as neighbors. The more neighbors we consider, the better features we calculate, enhancing a model’s performance. However, considering a lot of points as neighbors increase the computational complexity, requiring a high configuration computing device. Additionally, for a higher radius value, points can be selected from different objects or areas, which may increase the computational complexity and decrease the accurate segmentation performance. Therefore, the selection of an optimal radius is important.

#### 3.2.2 Adaptive neighborhood selection.

Given the inherent complexity and heterogeneity of LiDAR point cloud data, feature values in this study are also computed using an adaptive neighborhood selection strategy. Specifically, we employ the adaptive neighborhood selection technique proposed by Chakraborty et al. [1], which automatically adjusts the spatial context based on local point density and distribution characteristics. To choose an appropriate neighborhood, the method divided the input point clouds into four different regions such as the planar region, the vertical region, the low omnivariance region, and the high omnivariance region.

The input point cloud is initially divided into regular and scattered regions based on the curvature value at each point. The regular region is further classified into planar (e.g., rooftops, ground) and vertical (e.g., facades, poles) subregions using the computed verticality of individual points. Meanwhile, the scattered region is subdivided into low and high omnivariance areas based on the omnivariance values of the points within this region.

For neighborhood selection, distinct methods are applied adaptively depending on the region type:

- An entropy-based neighborhood selection method [45] is employed for both planner and vertical regular regions.
- A normal direction-based neighborhood selection approach [44] is adopted for low omnivariance areas (e.g., building edges).
- For high omnivariance regions, which include vegetation, shrubs, and outliers, a Persistent Homology-based simplicial complex neighborhood selection method [46] is applied.

Finally, feature values for individual points are computed based on their respective neighborhoods, as determined by the region-specific selection method.

### 3.3 Features

Point cloud datasets primarily consist of 3D coordinates; sometimes, additional information, such as RGB color information, is augmented. However, it is the carefully calculated, suitable geometric features that significantly enhance the robustness of machine learning models. To compute these features for a given point, a support region must be defined first. In our experiments, we used both fixed-radius and adaptive neighborhood selection methods to define this region, aiming to ensure robustness of the experiments. The geometric features are then derived using each neighborhood selection method from the covariance matrix.

In a three-dimensional space, for any point $P_{i}(x,y,z)$, the covariance matrix is:


$$\begin{array}{c} Cov(x,y,z)=[\begin{array}{ccc} \text{Cov}(x_{i},\bar{x}) & \text{Cov}(x_{i},\bar{y}) & \text{Cov}(x_{i},\bar{z})\\ \text{Cov}(y_{i},\bar{x}) & \text{Cov}(y_{i},\bar{y}) & \text{Cov}(y_{i},\bar{z})\\ \text{Cov}(z_{i},\bar{x}) & \text{Cov}(z_{i},\bar{y}) & \text{Cov}(z_{i},\bar{z}) \end{array}] \end{array}$$ (2)

where $\text{Cov}(x_{i},\bar{x})$ is the variance of the x-coordinates (diagonal) and $\text{Cov}(x_{i},\bar{y})$ is the Covariance between the *x* and *y* coordinates (off diagonal). If *x* and *y* are two coordinates, then the covariance is calculated as follows,


$$\begin{array}{c} \text{Cov}(x,y)=\frac{1}{N-1}\sum_{i=1}^{N}(x_{i}-\bar{x})(y_{i}-\bar{y}) \end{array}$$ (3)

where *N* is the total number of data points in the selected neighborhood. *x*_*i*_ and *y*_*i*_ are any points in *N*. $\bar{x}$ and $\bar{y}$ are the means of all *x* and *y* points, respectively.

From the covariance matrix *Cov*(*x*,*y*,*z*) and identity matrix *I*_3×3_, we calculate three eigenvalues $\lambda_{i}$, where $\lambda_{1}>\lambda_{2}>\lambda_{3}$. However, points with fewer than three neighboring points may produce fewer eigenvalues; in such cases, missing values are treated as zero to maintain consistent dimensionality. After fetching the eigenvalues, we calculate the following eigenvalue-based features:


$$\begin{array}{c} \text{Sum of Eigenvalues}=\lambda_{1}+\lambda_{2}+\lambda_{3} \end{array}$$ (4)

$$\begin{array}{c} \text{Linearity}=(\lambda_{1}-\lambda_{2})/\lambda_{1} \end{array}$$ (5)

$$\begin{array}{c} \text{Planarity}=(\lambda_{2}-\lambda_{3})/\lambda_{1} \end{array}$$ (6)

$$\begin{array}{c} \text{Sphericity}=\lambda_{3}/\lambda_{1} \end{array}$$ (7)

$$\begin{array}{c} \text{Omnivariance}=\sqrt[{3}]{\lambda_{1}\lambda_{2}\lambda_{3}} \end{array}$$ (8)

$$\begin{array}{c} \text{Anisotropy}=(\lambda_{1}-\lambda_{3})/\lambda_{1} \end{array}$$ (9)

$$\begin{array}{c} \text{Eigenentropy}=\sum_{i=1}^{3}\lambda_{i}\ln(\lambda_{i}) \end{array}$$ (10)

$$\begin{array}{c} \text{Surface Variation}=\lambda_{3}/(\lambda_{1}+\lambda_{2}+\lambda_{3}) \end{array}$$ (11)

$$\begin{array}{c} \text{Surface Curvature}=\frac{\lambda_{3}}{\lambda_{1}+\lambda_{2}+\lambda_{3}} \end{array}$$ (12)

$$\begin{array}{c} \text{Verticality}=1-\lambda_{3}/(\lambda_{1}+\lambda_{2}+\lambda_{3}) \end{array}$$ (13)

$$\begin{array}{c} \text{Gaussian Curvature}=\lambda_{1}\lambda_{2} \end{array}$$ (14)

Along with the above eigenvalue-based features, the following geometric features are also considered.


$$\begin{array}{c} \text{Roughness}=\sigma_{Z}=\sqrt{\frac{1}{N}\sum_{i=1}^{N}(Z_{i}-\bar{Z}{)}^{2}} \end{array}$$ (15)

$$\begin{array}{c} \text{Mean Curvature}=\frac{\lambda_{1}+\lambda_{2}}{2} \end{array}$$ (16)

$$\begin{array}{c} \text{Normal Change Rate}=\frac{1}{N}\sum_{i=1}^{N}||{𝐧}_{i}-𝐧|| \end{array}$$ (17)

$$\begin{array}{c} \text{Number of Neighbors}=N \end{array}$$ (18)

$$\begin{array}{c} \text{Local Surface Normal Variance}=\frac{1}{N}\sum_{i=1}^{N}||{𝐧}_{i}-\bar{𝐧}|{|}^{2} \end{array}$$ (19)

$$\begin{array}{c} \text{Volume Density}=\frac{N}{\frac{4}{3}\pi r^{3}} \end{array}$$ (20)

$$\begin{array}{c} \text{Height}=Z \end{array}$$ (21)

where $\sigma_{Z}$ denotes the standard deviation of the height values within a neighborhood. The vector **n**_*i*_ represents the normal vector of the *i*-th neighboring point, while **n** is the normal vector of the central point. The average normal vector of all neighboring points is represented by $\bar{𝐧}$. The variable *r* refers to the search radius used for volume density calculation.

The spectral feature, also known as color information, of each point is also incorporated in experiments for the dataset that contains it. These features are represented as Red, Green, and Blue (RGB) intensity values.

These feature sets are used for the base learner’s initial model training. The following Section 3.4, provides a detailed discussion on the classifiers employed in this study.

### 3.4 Classifiers (base lerners)

Five machine learning classifiers: Random Forest (RF), Extreme Gradient Boosting (XGBoost), Light Gradient Boosting Machine (LightGBM), Categorical Boosting (CatBoost), and Adaptive Boosting (AdaBoost) are used as base learners in this study. These models are selected due to their proven effectiveness in handling high-dimensional and non-linear data structures, making them suitable for geometric feature-based classification tasks [47–51]. Among these models, RF is a bagging method, and the other four are boosting methods. Bagging is an ensemble learning method that reduces variance by training multiple models independently on different random subsets of the training data created by bootstrapping and then combining their predictions through averaging (for regression) or majority voting (for classification). On the other hand, boosting is an ensemble learning method that reduces both bias and variance by sequentially training models, where each model focuses on correcting the errors made by the previous models, and the final prediction is a weighted combination of the predictions of all models.

#### 3.4.1 Random Forest (RF).

Random Forest generates a large number of decision trees that make their predictions. A decision tree is a supervised prediction algorithm having a tree-like structure that recursively splits the data based on random feature values of the data, where each node represents a feature, each branch gives an outcome, and the leaf node represents the final value [52]. For a classification task, the RF uses prediction voting. The label with the most votes is selected as the final label. Generating multiple trees may create an overfitting issue; however, prediction voting solves this problem. The more decision trees RF generates, the better results it produces.

#### 3.4.2 Extreme Gradient Boosting (XGBoost).

XGBoost (Extreme Gradient Boosting) [53] is an optimized gradient-boosting algorithm designed for computational efficiency and high predictive accuracy. It employs an ensemble of sequentially trained decision trees, where each subsequent tree corrects errors from previous iterations by minimizing a differentiable loss function. The model fine-tunes predictions with gradients and second-order derivatives, resulting in exact updates. XGBoost uses advanced approaches such as shrinkage (learning rate modification), row and feature subsampling, and regularization (L1 and L2) to reduce overfitting and improve generalization. Unlike the Random Forest, it generates the final output by combining tree forecasts using a weighted summation. The number of boosting rounds is an important parameter, and careful adjustment is required for optimal performance. Despite its computational complexity, XGBoost’s scalability via parallel processing and ability to manage missing data make it one of the most popular algorithms for structured data processing.

#### 3.4.3 Light Gradient Boosting Machine (LightGBM).

LightGBM is a high-performance gradient boosting framework specifically designed to handle large datasets with high dimensionality efficiently [54]. It is also based on decision trees and operates by constructing an ensemble of weak learners to iteratively minimize the loss function. Unlike traditional gradient boosting methods, LightGBM uses a unique leaf-wise tree growth strategy instead of a level-wise approach, allowing it to grow trees by splitting the leaf with the maximum loss reduction at each step. This approach results in deeper and more complex trees that enhance model accuracy while maintaining efficiency.

For a dataset *D* = (*x*_*i*_, *y*_*i*_) where *x*_*i*_ are the features and *y*_*i*_ are the target values, the output of the model is given as the sum of predictions from *T* additive trees:


$$\begin{array}{c} \hat{y_{i}}=\sum_{t=1}^{T}f_{t}(x_{i}), \end{array}$$ (22)

where *f*_*t*_ represents the prediction from the *t*-th tree. *f*_*t*_ typically depends on *t*he tree structure.

LightGBM also supports advanced features like categorical feature handling, monotonic constraints, and parallel training, making it highly versatile and efficient. Its scalability, faster training, and lower memory usage make it an excellent choice for large-scale machine learning problems, outperforming many traditional gradient boosting frameworks in terms of speed and accuracy.

#### 3.4.4 Categorical Boosting (CatBoost).

CatBoost is a high-performance gradient boosting algorithm designed to effectively handle datasets with both numerical and categorical features [55]. It is based on decision trees and builds an ensemble of weak learners to minimize a loss function iteratively. Unlike other boosting algorithms, CatBoost processes categorical features natively without requiring preprocessing techniques like one-hot encoding, making it highly efficient and memory-effective.

CatBoost introduces two key innovations: First, ordered boosting, which prevents target leakage by training each model only on preceding instances, ensuring unbiased predictions; and second, statistics-based categorical encoding, which converts high-cardinality features into numerical values via conditional probabilities, enhancing robustness. The model also employs a symmetric tree structure, where all branches split on the same feature and threshold. This design improves computational efficiency, speeds up inference, and reduces overfitting by maintaining uniformity in tree growth.

CatBoost’s ability to handle missing data, imbalanced datasets, and categorical features natively, combined with its efficient computation and strong regularization techniques, makes it ideal for classification, regression, and ranking tasks. It’s easy to use and requires minimal hyperparameter tuning.

#### 3.4.5 Adaptive Boosting (AdaBoost).

AdaBoost (Adaptive Boosting) is one of the earliest and most influential ensemble learning methods, designed to improve the performance of weak learners by combining them into a strong predictive model [56]. AdaBoost utilizes decision stumps (single-level decision trees) as weak learners, achieving strong predictive performance through iterative reweighting of misclassified instances. This adaptive weighting mechanism enables effective handling of both classification and regression tasks by concentrating modeling effort on challenging cases.

During training, the algorithm assigns weights to all training instances. Initially, all samples are weighted equally, but after each iteration, the weights of misclassified samples are increased, directing the next learner’s focus toward those harder cases. This adaptive process continues until a specified number of learners are added or performance plateaus. Each weak learner contributes to the final prediction with a weight that reflects its accuracy.

Due to its conceptual simplicity, strong theoretical foundation, and practical effectiveness, AdaBoost continues to be a widely used algorithm in machine learning, serving as a benchmark and a foundational concept for more advanced ensemble methods such as Gradient Boosting Machines.

### 3.5 Stacked ensemble machine learning architecture

A Stacked Ensemble Machine Learning architecture is finally employed in this study to enhance the segmentation performance of LiDAR point cloud data. This approach combines multiple base machine learning models to leverage their individual strengths, ultimately improving the accuracy and robustness of the segmentation process. The proposed ensemble in this study consists of five base models as described in Section 3.4 with Logistic Regression serving as the meta-model. Logistic Regression is a statistical model used for segmentation tasks, where it predicts the probability of an outcome based on one or more predictor variables. It works by applying the logistic function (sigmoid) to a linear combination of input features, transforming the output into a probability score between 0 and 1. In this setup, the logistic regression calculates the probability of each class independently, and the class with the highest probability is chosen as the final prediction. This allows the model to handle multiple classes efficiently while maintaining its simplicity and interpretability in an ensemble setting.

**Fig 3:**
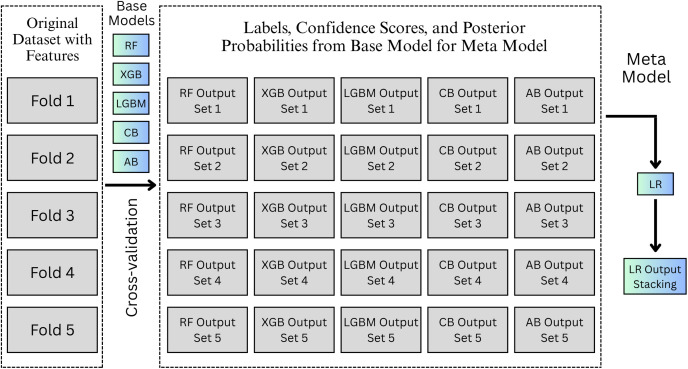
Stacking ensemble learning model framework.

The process begins by independently training each base model on the training data. The training data are divided into five folds as shown in Fig 3 because this architecture leverages the cross-validation k-fold. Each model generates predictions, which are then fed into the meta-model. The meta-model (Logistic Regression), learns from the outputs of these base models and makes the final segmentation decision. This allows the meta-model to optimize the combination of predictions from the base models, improving the overall performance of the system.

The stacked ensemble architecture benefits from the strengths of the different base models. For example, RF captures complex feature interactions and provides stability, XGB offers powerful error correction and regularization, LGBM is efficient for handling large datasets, CB excels in handling categorical features, and AB is adept at boosting weak learners’ performance. By combining these models, the ensemble reduces the likelihood of overfitting, enhances model generalization, and produces more accurate segmentation results.

This stacking method not only mitigates the individual weaknesses of each base model but also ensures a more robust performance across a variety of scenarios, including high-dimensional, noisy, and complex data. The meta-model’s ability to refine predictions from diverse base models allows the ensemble to achieve superior segmentation accuracy compared to the individual base models.

### 3.6 SHAP analysis

Shapley additive explanation (SHAP) is an XAI approach that explains the importance, relevance, and relationship between input features [37]. It enhances the interpretability of the black box machine learning model by quantifying and ranking the contribution of individual features to segmentation outcomes, revealing how each feature influences predictions.

There are different variants of SHAP, such as TreeSHAP [39], KernelSHAP [57], and LinearSHAP [37]. TreeSHAP is designed for calculating feature-importance and interoperability in tree-based machine learning models, which mainly use a linear explanatory model and Shapley values for prediction [39]. Thus, this study used TreeSHAP for Random Forest, XGBoost, LightGBM, and CatBoost classifiers. KernelSHAP calculates the importance of each feature by approximating the Shapley values using a weighted linear regression model. It can interpret without modifying any model. Thus, this study used the KernelSHAP to check the interpretability of the AdaBoost classifier. Additionally, LinearSHAP was applied to the Logistic Regression model, also known as meta-lerner in the architecture. LinearSHAP computes Shapley values for linear models by assuming input feature independence, where the Shapley value for each feature is approximated with the intercept, derived from the model’s weight coefficients.

Although different SHAP algorithms operate differently depending on the model used, they all follow a unified mathematical framework to calculate the Shapley values. The general equation for computing Shapley values is shown below.


$$\begin{array}{c} \phi_{i}=\sum_{S_{f}\subseteq N_{f}⧵\{i\}}\frac{|S_{f}|!(N_{f}-|S_{f}|-1)!}{N_{f}!}\left[f(S_{f}\cup \{i\})-f(S_{f})\right] \end{array}$$ (23)

The Shapley value $\phi_{i}$ for any feature *i* quantifies the contribution of that feature to the model’s prediction. Let *N*_*f*_ represent the set of all features, and *S*_*f*_ denote a subset of *N*_*f*_ that excludes the feature *i*. The model’s prediction using only the features in *S*_*f*_ is given by *f*(*S*_*f*_), while $f(S_{f}\cup \{i\})$ represents the prediction when the feature *i* is also included in the set. Thus $\left[f(S_{f}\cup \{i\})-f(S_{f})\right]$ measures how much feature *i* changes the prediction when added to subset *S*_*f*_. The coefficient $\frac{|S_{f}|!(N_{f}-|S_{f}|-1)!}{N_{f}!}$ ensures fair weighting across all possible feature combinations. The Shapley value averages the marginal contributions of feature *i* across all possible subsets of features.

### 3.7 Evaluation metrics

The results of this study are evaluated using Accuracy, Precision, Recall, and F1-score. Accuracy depicts the proportion of correctly predicted points of all points. True Positive (TP) is the number of points where the model predicted label is positive and the ground truth label is also positive. True Negative (TN) is the same as TP, where both the model predicted and the ground truth label are negative. False Positive (FP) is the number of points that the model predicts as positive, but the ground truth is negative. Vice versa, False Negative (FN) is the number of points that the model predicts as negative, but the ground truth is positive. So, correctly estimated values are TP and TN; whereas, incorrectly estimated values are FP and FN. The equations of the four evaluation metrics are shown in the following:


$$\begin{array}{c} \text{Accuracy}=\frac{\text{TP}+\text{TN}}{\text{TP}+\text{FP}+\text{TN}+\text{FN}} \end{array}$$ (24)

$$\begin{array}{c} \text{Precision}=\frac{\text{TP}}{\text{TP}+\text{FP}} \end{array}$$ (25)

$$\begin{array}{c} \text{Recall}=\frac{\text{TP}}{\text{TP}+\text{FN}} \end{array}$$ (26)

$$\begin{array}{c} \text{F1-Score}=2\times \frac{\text{Precision}\times \text{Recall}}{\text{Precision}+\text{Recall}} \end{array}$$ (27)

For Precision, Recall, and F1-Score, both the weighted average and macro average is considered for evaluation. The weighted average calculates the average of a metric based on the support (number of instances in each class) of each class. Whereas, macro average calculates the average of a metric by giving equal weights to each class, which does not consider class imbalance.

The weighted average reflects the dominance of frequent classes in real-world problems. It prevents a rare class from skewing the overall metric. On the other hand, the macro average focuses on the performance of a rare but critical class, as it reveals the struggle of a model with a minority class.

## 4 Experiments

### 4.1 Experimental setup

The experiments are conducted utilizing the Python programming language. We employed Principal Component Analysis (PCA) from the Scikit-learn library’s decomposition module to compute geometric features of the point data, while additional features were derived through manual calculations. The same libraries ensemble module are used to implement Random Forest and AdaBoost machine learning classifiers. The other three classifiers, XGBoost, LightGBM, and CatBoost, have their libraries with their names. The StackingClassifier class from the ensemble module of the Scikit-learn library was used to implement the stack ensemble learning architecture. The set-up of our experiment comprises of a 4th-generation Intel Xeon processor with a clock speed of 2.20 GHz, 128 GB of DDR4 RAM, a 16 GB NVIDIA RTX A4000 GPU with 6144 CUDA cores, and the Ubuntu 22.04.4 operating system.

### 4.2 Hyperparameter tuning

Hyperparameter tuning refers to the process of identifying the optimal combination of hyperparameters that minimizes the loss function, thereby enhancing model accuracy and generalization. To achieve optimal predictive performance from machine learning models, it is essential to determine the most suitable hyperparameters for the given dataset. Hyperparameters play a crucial role in regulating the learning process of an ML model. For hyperparameter selection we used the Grid Search technique for each of the models. We defined a set of parameters in a grid. The grid search exhaustively searches all possible combinations of hyperparameters and provides the best possible set of hyperparameters. For the Random Forest model the n_estimators, max_depth, max_features are used as ‘200’, ‘None’, and ‘Auto’, respectively. For the XGBoost model use_label_encoder, eval_metric, booster, n_estimators are used as ‘False’, ‘mlogloss’, ‘gbtree’, and ‘100’, respectively. For the LightGBM model num_leaves, n_estimators, and learning_rate are used as ‘31’, ‘100’, ‘-1’, and ‘0.1’, respectively. For the CatBoost model learning_rate, loss_function, and eval_metric are used as ‘0.1’, ‘MultiClass’, and ‘Accuracy’, respectively. Lastly, for the AdaBoost model n_estimators, estimator, learning_rate, and algorithm are used as ‘100’, ‘base’, ‘0.5’, and ‘SAMME’, respectively. Additionally, for all the models random_state are used as ‘42’. For the Logistic Regression model that was used a the meta model, penalty, and max_iter are used as ‘l2’, and ‘100’, respectively.

### 4.3 Support area selection

The area surrounding a point, considered to select neighbors, is called the support area. The radius taken for the support area is called the support radius *r*. To select the neighboring points for feature value calculation, this study empirically selects a support area that gives the best segmentation performance for the individual model. Table 1 demonstrates the accuracy of individual base models using both Fixed and adaptive neighborhood selection techniques for different support areas. All features described in Section 3.3 are used for segmentation.

**Table 1:** Accuracy comparison of base classifiers on two datasets with different fixed neighborhood radius and adaptive neighborhood radius.

Classifiers	Accuracy (%)											
	Vaihingen Dataset (5 points/m^2^)						Toronto3D Dataset (1000 points/m^2^)					
	Fixed Neighborhood Radius			Adaptive Neighborhood Radius			Fixed Neighborhood Radius			Adaptive Neighborhood Radius		
	1m (5pts)	2m (20pts)	3m (45pts)	1m	2m	3m	0.05m (2.5pts)	0.1m (10pts)	0.2m (40pts)	0.05m	0.1m	0.2m
**Random Forest**	83.35	86.35	88.62	82.86	83.60	83.94	83.93	88.88	92.17	94.78	93.62	92.80
**XGBoost**	76.71	79.54	81.63	73.97	73.56	73.77	83.45	88.08	90.88	93.49	91.85	91.02
**LightGBM**	74.68	74.84	75.94	71.58	71.58	70.41	82.85	87.57	90.15	92.30	91.11	88.13
**CatBoost**	76.07	79.09	81.12	73.01	72.80	72.78	83.53	88.16	91.00	93.45	91.85	91.07
**AdaBoost**	83.44	87.98	90.68	83.37	84.08	84.61	80.25	84.73	89.71	91.58	89.69	88.55

For fixed radius neighborhood selection, an optimum support radius is a must, which is time-expedient, resource-efficient, and also quantifiable. For the Vaihingen dataset, we take 1 m, 2 m, and 3 m radius that considers approximately 5 points, 20 points, and 45 points, respectively. For the Toronto3D dataset, we take 0.05 m, 0.1 m, and 0.2 m radius that corresponds to 2.5 points, 10 points, and 40 points, respectively. The number of points considered in a support area is tried to keep similar so that the impact of density in datasets is minimized. As we see, the more points we consider, the better segmentation accuracy it provides. While considering the adaptive neighborhood selection method, we divided the point cloud into four different regions based on curvature, verticality, and omnivariance.

Table 1 demonstrates that the Vaihingen dataset exhibits maximum accuracy for *r* = 3m, and the Toronto3D dataset exhibits the best output for *r* = 0.2m when using the Fixed Neighborhood radius. Additionally, for the Adaptive Neighborhood, the Vaihingen dataset exhibits maximum accuracy for the same radius as in the fixed case, but the Toronto3D dataset performs best at *r* = 0.05m. Thus, these optimal values will be consequently employed in all subsequent experiments of this research.

### 4.4 Results and discussions

**Fig 4:**
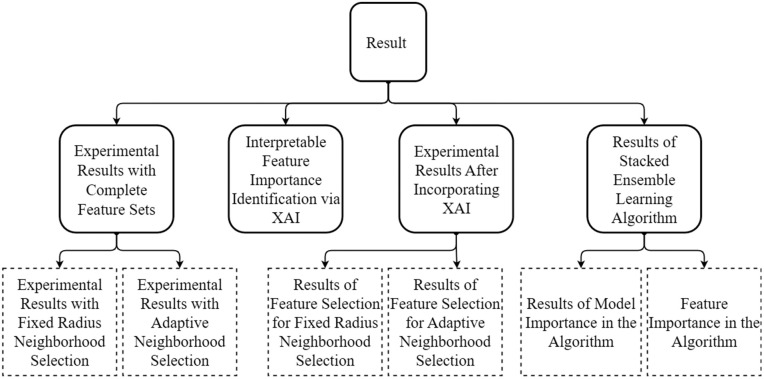
Organization of the Results Section.

This Section is organized as shown in Fig 4. Section 4.4.1 demonstrates the performance of individual base models using all the features described in Section 3.3. Section 4.4.2 illustrates the importance of individual features in each model. Section 4.4.3 tries to find the optimum feature sets using feature reduction based on XAI.

#### 4.4.1 Experimental Results with Complete Feature Sets.

Tables 2 and 3 show the performance of various models trained with fixed-radius neighborhood-based feature values on the Vaihingen dataset and Toronto3D dataset, respectively. For Vaihingen total 18 features described in Section 3.3 are used, and for Toronto3D additional 3 features, i.e., RGB values, are also used for training. AdaBoost achieved the highest F1 scores and accuracy scores on the Vaihingen dataset, while the Random Forest classifier outperformed on the Toronto3D dataset across all performance metrics.

**Table 2:** Performances of Different Classifiers with 18 Geometric Features for Vaihingen Dataset with Fixed Neighbourhood Radius.

Classifiers	Weighted Average			Macro Average			Accuracy
	Precision	Recall	F1	Precision	Recall	F1	
Random Forest	88.73	88.62	88.41	83.52	72.05	76.55	88.62
XGBoost	81.94	81.63	81.45	78.95	68.80	72.87	81.63
LightGBM	76.47	75.94	75.62	63.69	57.15	59.29	75.94
CatBoost	81.40	81.12	80.88	78.06	65.15	69.93	81.12
AdaBoost	90.63	90.68	90.62	84.36	79.72	81.85	**90.68**

**Table 3:** Performances of Different Classifiers with 21 Features for Toronto3D Dataset with Fixed Neighbourhood Radius.

Classifiers	Weighted Average			Macro Average			Accuracy
	Precision	Recall	F1	Precision	Recall	F1	
Random Forest	92.12	92.17	91.97	88.20	74.63	79.60	**92.17**
XGBoost	90.76	90.88	90.65	83.99	71.90	76.39	90.88
LightGBM	89.97	90.15	89.90	77.00	69.36	72.56	90.15
CatBoost	90.89	91.00	90.77	84.67	72.09	76.77	91.00
AdaBoost	89.50	89.71	89.51	78.28	70.00	73.42	89.71

Tables 4 and 5 demonstrate the performance metrics of various models using Adaptive radius-based feature values on the Vaihingen dataset and Toronto3D dataset, respectively.

**Table 4:** Performances of different classifiers using Adaptive Neighborhood based 18 Geometric Features on the Vaihingen dataset.

Classifiers	Weighted Average			Macro Average			Accuracy
	Precision	Recall	F1	Precision	Recall	F1	
Random Forest	83.85	83.94	83.75	79.56	68.24	72.51	83.94
XGBoost	73.94	73.77	72.78	73.09	49.84	55.04	73.77
LightGBM	70.68	70.41	69.37	57.33	46.76	48.76	70.41
CatBoost	72.59	72.78	71.54	73.06	45.42	48.91	72.78
AdaBoost	84.46	84.61	84.52	74.91	70.96	72.75	**84.61**

**Table 5:** Performances of different classifiers using Adaptive Neighborhood based 21 Geometric Features on the Toronto3D dataset.

Classifiers	Weighted Average			Macro Average			Accuracy
	Precision	Recall	F1	Precision	Recall	F1	
Random Forest	94.73	94.78	94.63	91.99	81.26	85.77	**94.78**
XGBoost	93.37	93.49	93.31	86.47	77.87	81.56	93.49
LightGBM	92.08	92.30	92.07	75.67	69.17	71.94	92.30
CatBoost	93.31	93.45	93.25	85.69	76.86	80.65	93.45
AdaBoost	91.62	91.58	91.60	77.34	78.26	77.79	91.58

It can be noticed that for the Vaihingen dataset, Adaptive radius-based feature selection shows slightly less accuracy. This is because the dataset is less dense. Moreover, the distribution of points into the four regions is uneven. This resulted in a slightly less accurate feature calculation, leading to a lower accuracy in model prediction. However, the adaptive neighborhood performs better in the Toronto3D dataset in terms of the final F1 scores and accuracy. This is because the Toronto3D dataset is very dense; thus, the uneven distribution does not hamper the understanding of the object due to its density. However, Random Forest performs better on the Toronto3D dataset due to its ability to handle the high point density, while AdaBoost shows superior performance on the Vaihingen dataset, where the lower point density aligns better with its model characteristics.

#### 4.4.2 Interpretable feature importance identification via XAI.

XAI algorithms are implemented on trained models because they utilize a model’s trained parameters and algorithmic structure for explainability. Therefore, the trained models discussed in subsection 4.4.1 serve as the foundation for various XAI explainers. This explainer helps break down the model’s complex decision-making process into understandable contributions of each feature. The test data is then fit to the explainer to calculate SHAP values. These SHAP values show how much each feature pushes the model’s prediction higher or lower for each sample, helping interpret the impact of different features on every model’s decisions. The impact of features on each class is also identified.

**Fig 5:**
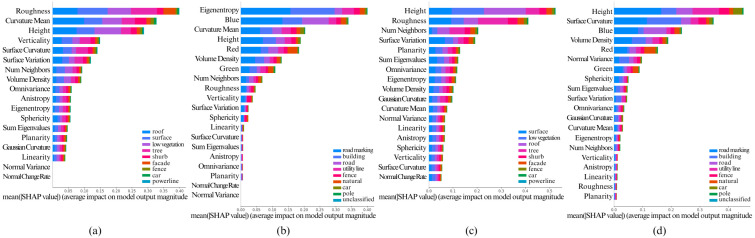
Calculated Feature SHAP values using TreeSHAP after classifying point cloud using RF classifier. **(a)** Fixed neighborhood-based SHAP feature values on Vaihingen, **(b)** Fixed neighborhood-based SHAP feature values on Toronto3D, **(c)** Adaptive neighborhood-based SHAP values on Vaihingen, and **(d)** Adaptive neighborhood-based SHAP values on Toronto3D.

**Fig 6:**
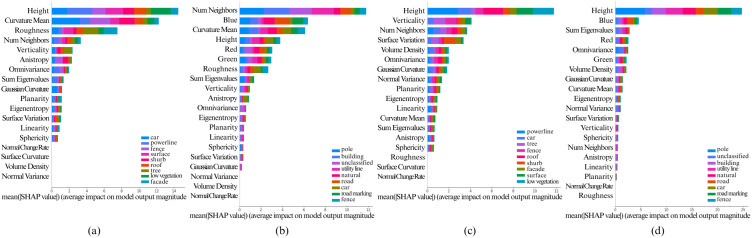
Calculated Feature SHAP values using TreeSHAP after classifying point cloud using XGBoost classifier. **(a)** Fixed neighborhood-based SHAP feature values on Vaihingen, **(b)** Fixed neighborhood-based SHAP feature values on Toronto3D, **(c)** Adaptive neighborhood-based SHAP values on Vaihingen, and **(d)** Adaptive neighborhood-based SHAP values on Toronto3D.

**Fig 7:**
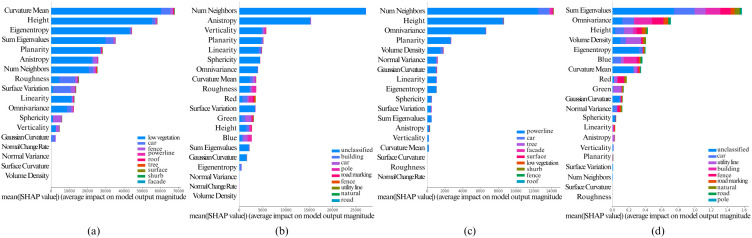
Calculated Feature SHAP values using TreeSHAP after classifying point cloud using LightGBM classifier. **(a)** Fixed neighborhood-based SHAP feature values on Vaihingen, **(b)** Fixed neighborhood-based SHAP feature values on Toronto3D, **(c)** Adaptive neighborhood-based SHAP values on Vaihingen, and **(d)** Adaptive neighborhood-based SHAP values on Toronto3D.

**Fig 8:**
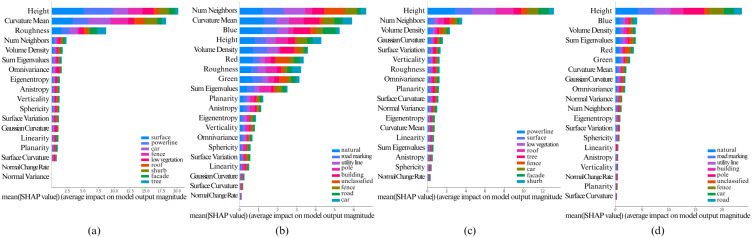
Calculated Feature SHAP values using TreeSHAP after classifying point cloud using CatBoost. **(a)** Fixed neighborhood-based SHAP feature values on Vaihingen, **(b)** Fixed neighborhood-based SHAP feature values on Toronto3D, **(c)** Adaptive neighborhood-based SHAP values on Vaihingen, and **(d)** Adaptive neighborhood-based SHAP values on Toronto3D.

**Fig 9:**
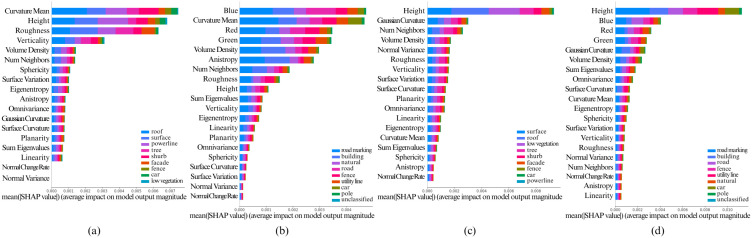
Calculated Feature SHAP values using KernelSHAP after classifying point cloud using AdaBoost. **(a)** Fixed neighborhood-based SHAP feature values on Vaihingen, **(b)** Fixed neighborhood-based SHAP feature values on Toronto3D, **(c)** Adaptive neighborhood-based SHAP values on Vaihingen, and **(d)** Adaptive neighborhood-based SHAP values on Toronto3D.

The Figs 5, 6, 7, 8, and 9 show the SHAP summary graphs for the Vaihingen and Toronto3D datasets for different base classifiers. The features are listed along the Y-axis, where the feature at the top has the most importance and the feature at the bottom has the least importance. Calculated SHAP importance values are represented along X-axis. Different classes are labeled with different colors. Every graph shows the 20 most important features.

Variations in the training data have minimal impact on the SHAP summary plots, as the feature importance rankings remain largely consistent. However, when model hyperparameters are varied, the top 6–10 most important features generally remain unchanged. That said, lower-impact features can sometimes shift in ranking due to hyperparameter adjustments. This is particularly true when the Shapley values of features are very close to each other, as slight changes in hyperparameters may cause these less significant features to be prioritized differently. Nonetheless, the most important features, particularly those with large differences in Shapley values, show stable rankings across different hyperparameter configurations. Overall, this indicates that the model’s key decision-making features are robust to variations in both data and hyperparameters.

#### 4.4.3 Experimental result after incorporating XAI.

The initially selected features (18 for Vaihingen and 21 for Toronto3D) were chosen based on insights from existing state-of-the-art literature. By applying the XAI algorithms, we aim to find out the optimal feature sets for the base learners to maintain the models’ effectiveness and efficiency. Considering this fact, we take the top 66%, 50%, and 33% of the initially selected features based on the SHAP-derived feature importance and observed the performances of various machine learning base classifiers accordingly. Thus, specifically, for the Vaihingen dataset, the top 12, 9, and 6 features are considered, while for the Toronto3D dataset top 14,11, and 9 features are considered for further evaluation.

Table 6 shows the results of the reduced feature sets for different classifiers on both Vaihingen and Toronto3D datasets. Neighborhoods are selected using the fixed radius neighborhood with a support area of 3m and 0.2m for Vaihingen and Toronto3D data, respectively. Because these support areas demonstrated the highest accuracy in Table 2 for the datasets. Table 7 shows similar experimental results in which the adaptive neighborhood selection technique was used to calculate the feature values for both datasets.

**Table 6:** Accuracy Comparison of Different Classifiers by Selected Feature Sets based on the Calculated Feature Importance by XAI. Fixed Radius Neighborhoods are Used for Calculating Feature Values.

Classifiers	Feature Selection for Fixed Radius Neighborhood							
	Vaihingen Dataset (3m radius)				Toronto3D Dataset (0.2m radius)			
	Percentage of Top Features			All Features (100%)	Percentage of Top Features			All Features (100%)
	66%	50%	33%		66%	50%	33%	
	Accuracy(%)							
**Random Forest**	89.02	89.72	82.38	88.62	92.36	92.49	89.80	92.17
**XGBoost**	81.54	81.09	79.89	81.63	90.87	90.83	89.90	90.88
**LightGBM**	75.08	73.67	69.07	75.94	90.14	88.82	77.76	90.15
**CatBoost**	80.66	80.55	77.24	81.12	91.00	91.00	89.49	91.00
**AdaBoost**	90.44	90.41	89.15	90.68	89.72	89.75	88.45	89.71

**Table 7:** Accuracy Comparison of Different Classifiers by Selected Feature Sets based on the Calculated Feature Importance by XAI. The Adaptive Neighborhood Selection Technique is Used for Calculating Feature Values.

Classifiers	Feature Value Calculated Using Adaptive Neighborhood Selection							
	Vaihingen Dataset (3m radius)				Toronto3D Dataset(0.05m radius			
	Percentage of Top Features			All Features (100%)	Percentage of Top Features			All Features (100%)
	66%	50%	33%		66%	50%	33%	
	Accuracy(%)							
**Random Forest**	84.87	85.69	85.25	83.94	94.46	94.69	94.69	94.78
**XGBoost**	72.82	72.61	71.60	73.77	92.90	93.32	93.41	93.49
**LightGBM**	69.97	70.19	68.25	70.41	88.73	92.09	92.40	92.30
**CatBoost**	71.86	71.69	70.74	72.78	92.58	93.29	93.41	93.45
**AdaBoost**	84.63	83.98	83.99	84.61	90.91	91.47	91.41	91.58

Table 6 shows that, in the Vaihingen Dataset, using all 18 features, on the calculated fixed radius-based feature value, the accuracy of RF is 88.62%; after selecting the top 66% features according to the XAI algorithm, the accuracy slightly increased to 89.02%. The accuracy even increased to 89.72% after taking the top 50% of the features. This phenomenon also occurred for the Toronto3D dataset, where the accuracy increased from 92.17% to 92.32% and 92.49% for the top 66% and 50% features, respectively. This indicates that the XAI-based algorithms help to improve the effectiveness. The training time also decreased for both datasets in every model. For the Vaihingen dataset, a reduction in the feature set resulted in a training time decrease of approximately 8% to 13% with each successive reduction in features. For the Toronto3D dataset, a reduction in the feature set led to a decrease in training time of approximately 6% to 8% with each successive feature reduction. This indicated the efficiency of the method. This occurred because some of the least significant features negatively impacted the decision-making process of the machine learning models.

**Fig 10:**
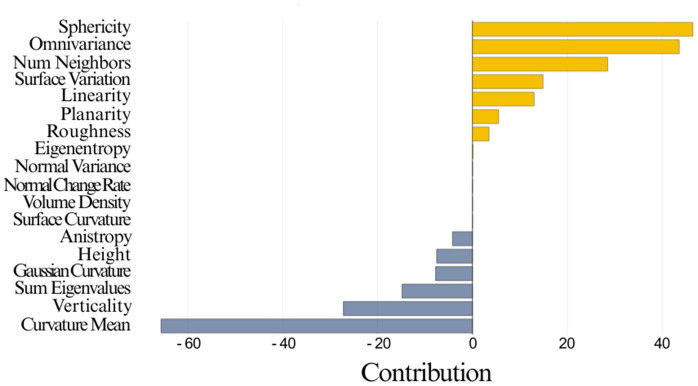
Local Interpretation of a point for the impact of different features on its prediction for the RF model.

To justify this issue, we consider the trained RF classifier on the Vaihingen dataset and choose a random point from the test set. The point belongs to the ‘tree’ class, and the classifier correctly classified it. Fig 10 shows the plotted shaply values for that point. The impact of each feature on the decision-making process of RF is shown here. The features below are negatively pulling RF from making the decision. Although the model predicted the point correctly, the confidence score was very low.

Table 7 also shows similar results as Table 6, feature values are calculated based on the adaptive neighborhood selection technique in Table 7. Performances increased for both 66% and 50% top features in the Vaihingen dataset. However, RF’s accuracy decreased when trained with the top 33% features. Thus, XAI suggests that the optimum number of features is between 50% and 33% of the features for the RF classifier. In some cases, fewer features show slightly less accuracy, which is negligible because of increased efficiency. The accuracy was preserved while reducing the dimensionality of features for the Toronto3D dataset.

The experimental results indicate that height and mean curvature are among the most influential geometric features in achieving high segmentation accuracy across multiple classifiers. Additionally, for datasets containing color information, color features also play a significant role in enhancing performance. Conversely, less significant features are normal change rate, planarity, and linearity.

In the following Section, we will demonstrate and discuss the results acquired through implementing the stacked ensemble learning. Additionally, we will find the importance of base models for the architecture and the feature importance for the whole architecture.

#### 4.4.4 Results of stacked ensemble learning algorithm.

This Section presents the results obtained through employing the stacked ensemble machine learning architecture described in Section 3.5. Both fixed-radius and adaptive neighborhood selection techniques were used to compute the original feature sets for the Vaihingen and Toronto3D datasets. Table 8 summarizes the results of the performance metrics for each dataset.

**Table 8:** Performances of Stacked Ensemble ML Classifier on two datasets of four situations.

Classifiers	Weighted Average			Macro Average			Accuracy
	Precision	Recall	F1	Precision	Recall	F1	
Stacked ML on Vaihingen with Fixed Neighborhood (3 Meter)	91.13	91.16	91.08	86.49	79.55	80.52	**91.16**
Stacked ML on Vaihingen with Adaptive Neighborhood (3 Meter)	86.41	86.47	86.33	81.03	71.55	75.24	**86.47**
Stacked ML on Toronto3D with Fixed Neighborhood (0.2 Meter)	93.27	93.39	93.20	86.96	74.88	79.31	**93.39**
Stacked ML on Toronto3D with Adaptive Neighborhood (0.05 Meter)	95.71	95.83	95.65	91.31	75.55	79.54	**95.83**

The stacked ensemble ML architecture consistently outperformed individual classifiers, achieving the highest segmentation accuracy across both datasets. For example, the ensemble method obtained an accuracy of 91.13% for Vaihingen (fixed radius) and 95.71% for Toronto3D (adaptive neighborhood), demonstrating the advantage of combining multiple models.

**Fig 11:**
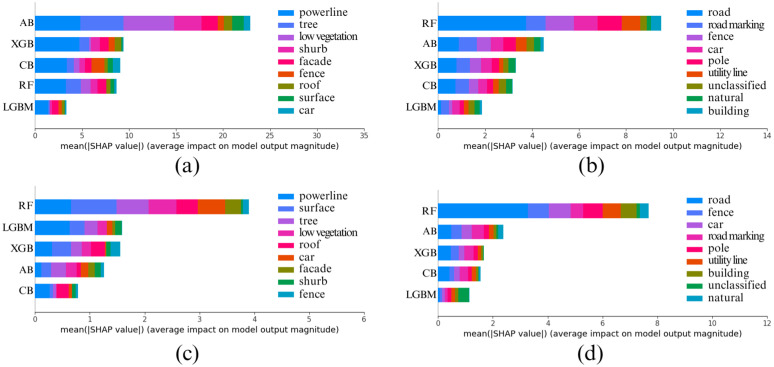
Importance of Base Models for Meta Model in Stacked Ensemble Learning Architecture that was used for segmentation task on (a) Fixed Neighborhood-based feature values on Vaihingen, (b) Fixed Neighborhood-based feature values on Toronto3D, (c) Adaptive Neighborhood-based feature values on Vaihingen, (d) Adaptive Neighborhood-based feature values on Toronto3D.

Now, we want to find which base models are most important for the architecture. We need to find out how accurately the prediction of individual base models matches the prediction of the whole architecture on test data. So, we extracted the predictions of each base model and stacked them, creating a new dataset. Then we passed the meta model and the new dataset to find the Shapley values. LinearSHAP treats the five base models as the features and treats the classes the same as before. By plotting those values, we get the importance of base models for this architecture. The Fig 11 shows the relative importance of each base model in the meta-model’s final prediction.

**Fig 12:**
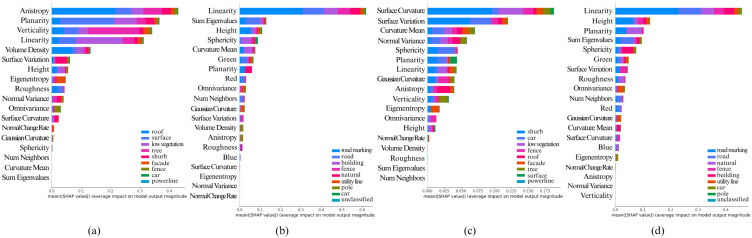
Calculated Feature SHAP values using KernelSHAP after classifying point cloud using Stacked Ensemble ML architecture for (a) Fixed Neighborhood-based feature values on Vaihingen, (b) Fixed Neighborhood-based feature values on Toronto3D, (c) Adaptive Neighborhood-based feature values on Vaihingen, (d) Adaptive Neighborhood-based feature values on Toronto3D.

Feature importance analysis using SHAP was also conducted to interpret the stack ensemble learning model’s decision-making process. The Fig 12 illustrates the SHAP summary plots, highlighting feature contributions to segmentation outcomes. The analysis revealed that features like height, mean curvature, and spectral information were crucial for high segmentation accuracy.

### 4.5 Quantitative Performance Comparison

Table 9 represents a quantitative performance evaluation of the proposed methods on the Vaihingen dataset. The proposed approach is compared with the state-of-the-art neighborhood selection methods of Chakraborty et al. [1], Nong et al. [42] and Xue et al. [20].

**Table 9:** Performance comparison for different methods on the Vaihingen dataset.

Method	Accuracy	Precision	Recall	F1 Score
Proposed	91.16	86.49	79.55	80.52
Chakraborty et al. [1]	91.30	87.34	78.42	82.89
Nong et al. [42]	85.72	72.77	77.80	75.13
Xue et al. [20]	79.56	72.90	57.39	64.27

Table 10 presents a quantitative performance evaluation of the proposed method on the Toronto dataset. The proposed approach is compared with the state-of-the-art methods of Chakraborty et al. [1], Sevgen et al. [49], and Huang et al. [58].

**Table 10:** Performance comparison for different methods on the Toronto3D dataset.

Method	Accuracy	Precision	Recall	F1 Score
Proposed	95.83	92.31	75.55	79.54
Chakraborty et al. [1]	96.13	89.44	76.39	78.55
Sevgen et al. [49]	95.10	78.93	87.67	82.33
Huang et al. [58]	81.27	–	–	45.10

### 4.6 Time complexity analysis

The Neighborhood Selection Method is integral to feature extraction in LiDAR point cloud segmentation and two primary methods of neighborhood selection are used in this study. In Fixed Radius Neighborhood Selection, the time complexity is directly related to the number of points and the radius, as for each point, the distance to all other points must be calculated. As such, the complexity is *O*(*n*^2^), where *n* is the number of points in the point cloud. This can be computationally expensive, especially in dense point clouds, due to the need to check all point pairs within the fixed radius. In Adaptive Neighborhood Selection the point cloud is divided into four regions: planar, vertical, low omnivariance, and high omnivariance and different neighborhood selection methods are applied to each region, such as entropy-based selection for planar and vertical regions, normal-distribution-based neighborhood selection for the low omnivariance regions, and a Persistent Homology-based approach for the high omnivariance regions. The time complexity of this method involves two major steps: pre-processing to classify points into regions, which has a complexity of *O*(*n*), and feature calculation, which, similar to the fixed-radius method, requires evaluating the neighborhood for each point. This results in a time complexity of *O*(*n*^2^) for the feature calculation step. Therefore, the overall complexity of the adaptive neighborhood selection method is dominated by the feature extraction and neighborhood selection steps, leading to a worst-case time complexity of *O*(*n*^2^), particularly when dealing with high-dimensional feature calculations.

The time complexity of the models in the stacked ensemble architecture plays a key role in the overall efficiency of the segmentation process. Random Forest (RF) has a time complexity of O(T·nlogn), where *T* is the number of trees and *n* is the number of data points, with parallelization improving efficiency. XGBoost also follows O(T·nlogn) due to sequential tree training, but optimizations like parallelization make it more efficient than traditional boosting methods. LightGBM uses a leaf-wise tree-growing strategy, leading to a faster training time of O(T·n), especially for large datasets. CatBoost, similar to XGBoost, has a complexity of O(T·nlogn), with additional efficiency gains from native categorical feature handling. Finally, AdaBoost has a complexity of O(T·n), with faster training due to the use of weak learners, like decision stumps. Each model’s complexity is influenced by the number of boosting rounds or trees, as well as the number of data points.

The time complexity of the methodology is mainly driven by the neighborhood selection methods and the models in the stacked ensemble. Both selection methods can be computationally expensive, especially for large datasets. The models, like Random Forest and XGBoost, add further complexity, but their optimization techniques can improve efficiency. Overall, the computational cost depends on the number of points, model parameters, and the selection process.

## 5 Conclusion

Developing an effective and efficient technique was the primary goal in LiDAR point cloud segmentation research. A major challenge in this regard was to select an appropriate and distinctive feature set for accurate machine learning-based segmentation. This research primarily addressed this issue by using explainable artificial intelligence (XAI) algorithms and found the importance of individual features, thereby enhancing the interpretability and performance of various black-box ML classifiers. Considering different explained machine learning classifiers as base models, this study proposed a stacked ensemble model to segment the LiDAR point cloud. The experimental results demonstrated that the stacked ensemble model outperformed the individual base models in segmenting the LiDAR point cloud of urban areas. Further, this study demonstrated the explainability of the ensembled model using different XAI algorithms.

Two types of LiDAR point cloud data, namely aerial and mobile LiDAR, were utilized in this study to conduct the experiments. It also investigated the influence of fixed-radius neighborhood and adaptive neighborhood selection approaches in computing the values of the geometric features for individual base models. Additionally, proper subsets of features were sampled based on their calculated importance to increase efficiency without compromising effectiveness.

This study demonstrated that reduced feature sets based on their importance enhance the effectiveness of each base model. Consequently, a reduced feature set decreased training and testing time, thereby directly enhanced the efficiency of the individual base model. Ultimately, the findings provided evidence that incorporating explainable artificial intelligence (XAI) can improve the overall performance of a stacked ensemble model for LiDAR point cloud segmentation.

However, this brings a crucial challenge, as it might lead to a potential data (feature) redundancy over the base models. Additionally, the mapping of features from base models to the meta-model poses a challenge, as each base model uses different features based on their importance, complicating the integration process within the stacked ensemble architecture. We also considered some non-linear models as meta-learners, but we rejected them because the computation of the Shapley values from explainers was very complicated and time-consuming. Moreover, our hardware resources were not sufficient to handle these large computations and store them. Addressing these issues will be a key focus of future research. Moreover, developing a computationally efficient variant of the proposed framework suitable for low-latency, real-time urban LiDAR applications will be an important direction for future investigation.
